# Multi-frequency ultrasound-assisted hot-air drying of *Cistanche deserticola* slices: Effects on drying efficiency, energy consumption, bioactive compounds, and microstructure

**DOI:** 10.1016/j.ultsonch.2026.108097

**Published:** 2026-10-08

**Authors:** Bowen Wu, Xiaopeng Huang, Guojun Ma, Fangxin Wan, Zepeng Zang, Yanrui Xu

**Affiliations:** College of Mechanical and Electrical Engineering, Gansu Agricultural University, Lanzhou 730070, China

**Keywords:** Cistanche, Multi-frequency ultrasound, Hot-air drying, Energy consumption, Bioactive compounds, Microstructure

## Abstract

This study systematically investigated the effects of single-frequency (SFU), dual-frequency (DFU), and triple-frequency ultrasound (TFU) combined with hot-air drying on the drying performance, energy consumption, physicochemical quality, characteristic bioactive compounds, and microstructure of Cistanche deserticola slices. All ultrasound treatments were conducted at 55 °C with a constant total ultrasonic power of 120 W using 25, 28, and 40 kHz individually or in combination. Ultrasound accelerated dehydration, with DFU and TFU reducing the drying time by up to 43.75% compared with conventional hot-air drying. Among the tested modes, DFU2 (25 + 40 kHz) showed the lowest specific energy consumption of 76.15 kWh/kg water, corresponding to a 32.28% reduction relative to hot-air drying. TFU was characterized by higher measured contents of phenolics, flavonoids, and polysaccharides, stronger antioxidant activity, lower color deviation, and generally higher contents of most characteristic phenylethanoid and iridoid glycosides. SEM observations showed that ultrasound produced a more open and porous tissue structure, consistent with the improved drying behavior. Multivariate analysis further distinguished the treatments, showing that TFU was more closely associated with most quality indicators, whereas DFU2 was more strongly associated with energy efficiency. These findings indicate that increasing the number of ultrasound frequencies did not result in uniform improvement across all responses; instead, different frequency modes favored different processing objectives. Overall, ultrasound-assisted HA improved drying performance while maintaining favorable quality attributes.

## Graphical abstract

**Figure:**
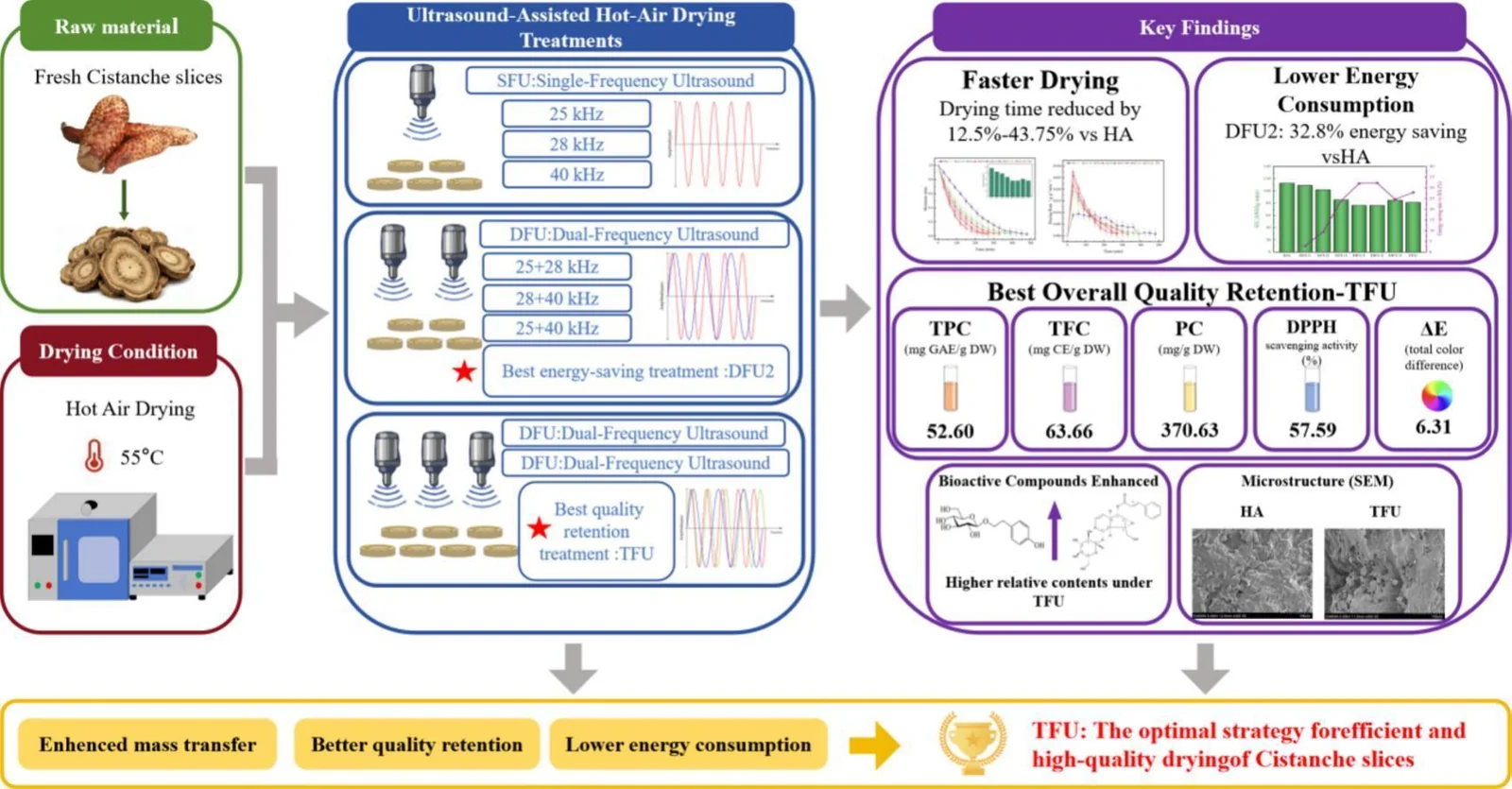

## 1 Introduction

*Cistanche deserticola* Ma is a parasitic member of Orobanchaceae and is widely used as a medicinal and edible botanical material in arid and semi-arid regions [1], [2]. It is rich in phenylethanoid glycosides, polysaccharides, iridoid glycosides, phenolics, flavonoids, and other bioactive compounds. Among them, echinacoside and acteoside/verbascoside are generally considered representative quality markers for Cistanche products [1], [3]. Because freshly harvested stems contain abundant water and remain metabolically active after harvest, quality deterioration can occur rapidly through browning, microbial spoilage, and degradation of labile constituents. Therefore, timely and appropriate dehydration is essential for extending shelf life, reducing transportation costs, and stabilizing product quality [4], [5], [6].

Among available dehydration techniques, hot-air drying (HA) remains attractive for industrial processing because the equipment is simple, scalable, and inexpensive to operate. Nevertheless, conventional HA commonly suffers from long drying times, high energy consumption, surface hardening, tissue shrinkage, color deterioration, and loss of thermolabile bioactive compounds [7], [8]. Technologies such as vacuum, microwave, radio-frequency, far-infrared, and pulsed-vacuum drying have improved the drying rate, color, rehydration capacity, shrinkage, and microstructure of Cistanche to varying degrees [4], [5], [6], [7], [9]. These findings indicate that the key objective in Cistanche drying is not merely rapid dehydration, but rather the promotion of moisture migration while minimizing thermal damage and maintaining the stability of characteristic bioactive compounds, color, and tissue structure. However, the industrial applicability of some advanced drying methods is still constrained by process complexity, energy characteristics, and equipment cost. Therefore, it is of clear engineering and practical significance to develop a coupled technology that can retain the industrial advantages of HA while markedly enhancing mass transfer and mitigating quality deterioration.

Power ultrasound provides mechanical energy to plant tissues during drying. The alternating acoustic pressure can disturb the liquid phase, deform cells periodically, and create localized shear near tissue surfaces. These effects help open transport pathways and weaken the resistance to water movement at both the sample surface and the internal matrix [10], [11]. When the acoustic field is properly matched with the material, drying time can be shortened and the loss of thermolabile compounds can be reduced. Reviews have reported that ultrasound, whether used as a pretreatment or as a synchronous drying assistance method, can shorten drying time, reduce energy consumption, and improve rehydration, microstructure, and retention of bioactive compounds [10], [12]. In medicinal and edible plants or high-moisture plant materials such as Cistanche, wolfberry, licorice, Codonopsis pilosula, cherry, and Chaenomeles sinensis, ultrasound-assisted drying or ultrasonic pretreatment has been shown to improve drying kinetics, microstructure, and quality attributes [13], [14], [15], [16], [17], [18]. However, ultrasound intensification does not always produce a positive response. Excessive ultrasonic power, prolonged treatment time, or inappropriate frequency may cause over-destruction of cellular structures, color deepening, non-enzymatic browning, or loss of bioactive compounds [19].

For Cistanche, a medicinal and edible botanical material, drying performance must be evaluated not only in terms of dehydration efficiency and physical quality but also with particular consideration of the preservation of characteristic phytochemicals, especially phenylethanoid and iridoid glycosides [1], [3]. Its high moisture content necessitates efficient dehydration, whereas prolonged thermal exposure or excessive ultrasonic disruption may adversely affect tissue integrity and labile bioactive constituents [7], [8], [19]. Therefore, ultrasound-assisted drying of Cistanche requires an appropriate balance between mass-transfer intensification and phytochemical-quality preservation, making the regulation of ultrasonic frequency mode particularly relevant.

In recent years, research on ultrasound-assisted drying has gradually shifted from conventional single- (SFU) toward dual- (DFU), multi-frequency ultrasound (MFU), and variable-frequency ultrasound systems. MFU can broaden the size response range of cavitation bubbles through the superposition of different acoustic frequencies, improve acoustic field uniformity, and enhance the continuity and spatial distribution of cavitation events, thereby producing a more uniform mass-transfer intensification effect. Fundamental studies in sonochemistry and bubble dynamics have demonstrated that dual- or triple-frequency acoustic fields can increase cavitation yield, lower the inertial cavitation threshold, and enhance mechanical effects through combination resonance and nonlinear bubble responses [20], [21], [22], [23], [24], [25]. In food processing, dual-frequency ultrasound (DFU) has been used to improve vacuum freeze drying of strawberry slices, while MFU ultrasound has been shown to promote the infrared drying of pineapple slices and improve quality attributes [26], [27]. Studies on freezing, gels, starch, and biopolymer systems have also indicated that MFU can improve food quality by regulating microstructure and water status [28], [29], [30], [31], [32], [33]. Applications of segmented variable-frequency ultrasound-assisted hot-air drying to rhubarb, Cistanche and cherry further demonstrate that frequency-mode regulation can influence drying rate, energy consumption, color retention, antioxidant activity, and overall quality [34], [35], [36]. These studies suggest that ultrasonic frequency mode is not merely an equipment parameter, but an important process variable that governs acoustic field distribution, mass-transfer behavior, tissue-structure evolution, and quality formation.

Accordingly, this study used hot-air drying as the basic drying method and synchronously applied three ultrasound frequencies (25, 28, and 40 kHz) in SFU, DFU, and TFU modes during the drying of Cistanche slices. The influence of each frequency combination on drying behavior, specific energy consumption, product quality, and microstructural characteristics was systematically evaluated. HCA and PCA were then used to integrate the quality and energy-related responses. By linking frequency mode with water removal, tissue opening, energy input, and phytochemical retention, this work seeks to identify ultrasound strategies suitable for efficient and low-damage drying of Cistanche and other medicinal and edible plant materials.

## 2 Materials and methods

### 2.1 Raw materials

Fresh Cistanche were obtained from Jinta County, Jiuquan, Gansu Province, China, in March 2026. Only intact samples with comparable maturity and size were used. Material showing visible decay, mildew, or mechanical injury was discarded. After surface cleaning, the selected stems were kept at 2–4 °C in a temperature- and humidity-controlled chamber before use. Based on the AOAC oven-drying method, the initial moisture content of the samples was determined after drying at 105 °C for 24 h and was 72.7% wet basis. Before each drying run, the samples were equilibrated for 1 h at 22 ± 1 °C, rinsed with distilled water, drained, peeled, and cut into 5 ± 0.5 mm slices. Each treatment used 120 ± 0.5 g of prepared slices.

### 2.2 Experimental equipment

HA was carried out in a YQ101-0A-4A electric forced-air oven (Beijing Yuqin Tengda Pharmaceutical Equipment Co., Ltd., China), operated at 220 V/50 Hz with a nominal power of 1.2 kW and an air velocity of 3 m/s. Ultrasound was supplied by a multi-frequency ultrasound-assisted hot-air drying system equipped with three ultrasonic transducer units and corresponding generators (THD-T1, Shenzhen Taiheda Technology Co., Ltd., China), covering frequencies of 25, 28, and 40 kHz. As shown in Fig. 1, the ultrasonic transducers were mounted beneath an ultrasonic vibration plate inside the drying chamber and were bonded to the underside of the plate. During drying, Cistanche slices were uniformly spread in a single layer on the upper surface of the vibration plate, allowing the samples to receive simultaneous hot-air and ultrasonic assistance. The hot air flowed through the chamber and across the sample layer, while ultrasonic vibration was transmitted from the transducers to the vibration plate and then to the samples.

**Fig. 1:**
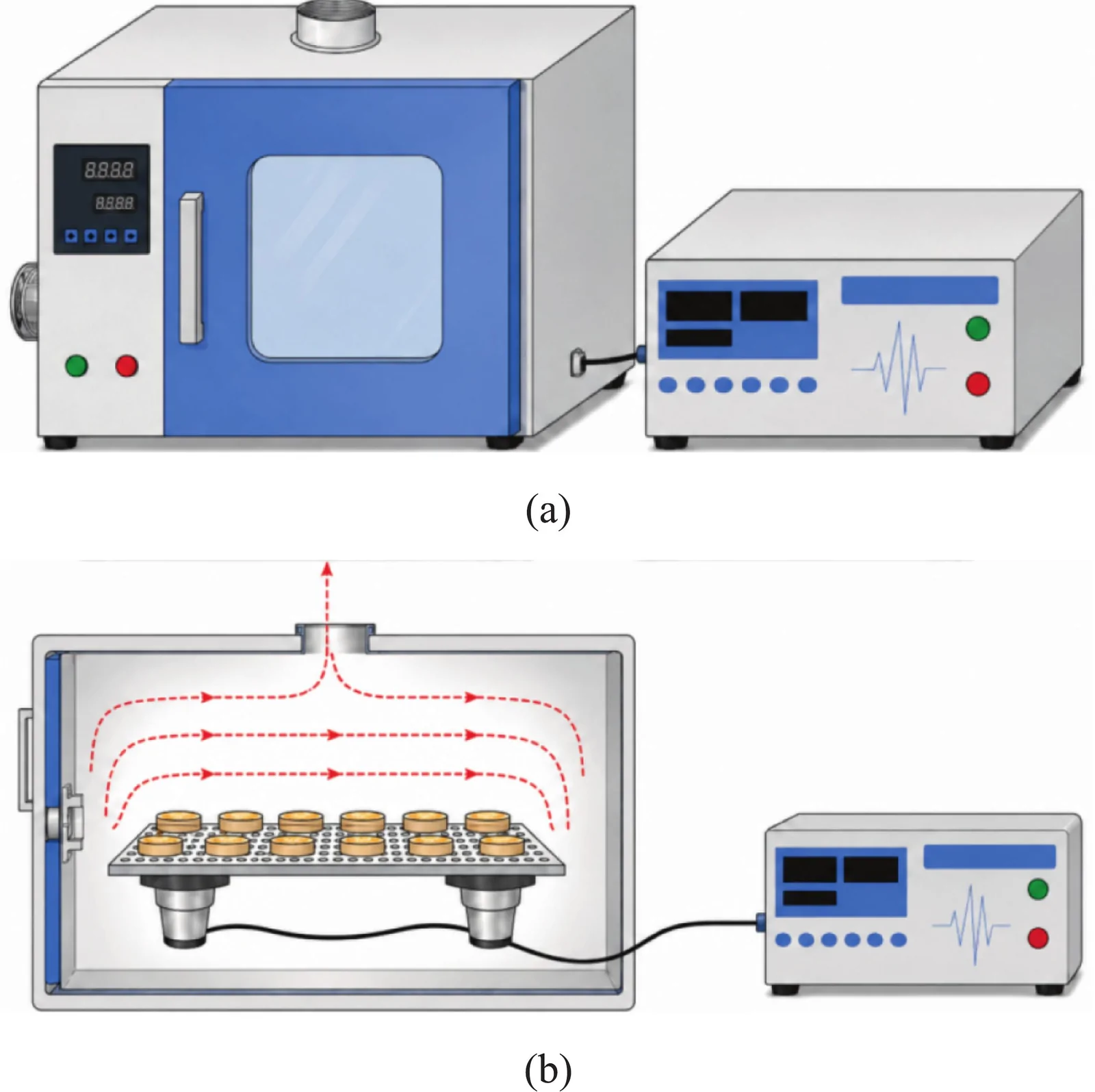
Schematic diagram of the multi-frequency ultrasound-assisted hot-air drying system. (a) External view; (b) Internal configuration of the drying chamber.

For the ultrasound-assisted treatments, SFU, DFU, and TFU modes were generated by activating the corresponding transducer units. The total ultrasonic power setting of the system was maintained at 120 W for all ultrasound-assisted treatments, and this total power was distributed among the activated transducers according to the selected frequency mode.

### 2.3 Drying treatments

#### 2.3.1 Control treatments

Natural drying (ND) and HA served as reference treatments. For HA, slices were arranged in one layer on trays and dried at 55 °C. Sample weight was measured every 30 min, and drying was continued until the moisture content decreased to below 10% on a wet basis. For ND, which was used as a traditional drying reference, slices of the same thickness were uniformly spread in a single layer on drying trays and placed near a window in a naturally ventilated indoor environment, where they were exposed to direct natural sunlight during the daytime with dust protection. During the ND period, the indoor temperature and relative humidity ranged from 17 to 22 °C and 34 to 39%, respectively. Samples were weighed every three days until the moisture content decreased below 10% on a wet basis.

#### 2.3.2 SFU-assisted HA

SFU-assisted HA was performed using HA as the basic drying method. Drying was carried out at a hot-air temperature of 55 °C, and the ultrasound output power was maintained at 120 W. During drying, 25, 28, and 40 kHz SFU treatments were synchronously applied until the same endpoint was reached. The three SFU treatments were denoted as SFU1, SFU2, and SFU3, respectively.

#### 2.3.3 DFU- and TFU-assisted HA

DFU- and TFU-assisted HA was also conducted at 55 °C with an ultrasonic power of 120 W. During drying, DFU at 25 + 28 kHz, 25 + 40 kHz, and 28 + 40 kHz, as well as TFU at 25 + 28 + 40 kHz, were synchronously applied until the same endpoint was reached. The frequency modes of each treatment are listed in Table 1.

**Table 1:** Frequency modes of different ultrasound-assisted hot-air drying treatments.

Treatment code	Frequency mode
SFU1	25 kHz
SFU2	28 kHz
SFU3	40 kHz
DFU1	25 + 28 kHz
DFU2	25 + 40 kHz
DFU3	28 + 40 kHz
TFU	25 + 28 + 40 kHz

### 2.4 Moisture ratio (MR), drying rate (DR) and effective moisture diffusivity(*D_eff_*)

The MR of Cistanche slices was calculated using Eq. (1):

(1) $$\mathrm{MR}=\frac{M_{t}-M_{e}}{M_{0}-M_{e}}$$

where *M_t_*, *M_0_*, and *M_e_* represent the moisture contents at time t, at the start of drying, and at equilibrium; and t is the time, min.

Because the equilibrium moisture content was negligible under the present conditions, the simplified form was used for data analysis as Eq. (2):

(2) $$\mathrm{MR}=\frac{M_{t}}{M_{0}}$$

The DR was calculated from the change in dry-basis moisture content over each sampling interval as Eq. (3):

(3) $$\mathrm{DR}=\;\frac{\Delta M}{\Delta t}$$

The *D_eff_* was estimated based on Fick’s second law by approximating the Cistanche slices as an infinite slab. Assuming a uniform initial moisture distribution and a constant effective moisture diffusivity, the calculation is expressed as Eq. (4):

(4) $$ln\left(MR\right)=ln\left(\frac{8}{\pi^{2}}\right)-\pi^{2}\frac{D_{\mathrm{eff}}}{{4L_{0}}^{2}}t$$

where *D_eff_* is the effective moisture diffusivity,m^2^/s; t is the drying time, s, and *L*_0_ is half of the initial slice thickness, m. Based on the nominal slice thickness of 5 mm, *L*_0_ was taken as 2.5 × 10^−3^ m. *D_eff_* was calculated from the slope (k) of the linear regression of ln(*MR*) versus drying time (Eq 5):

(5) $$D_{\mathrm{eff}}=\;\frac{{4L_{0}}^{2}k}{\pi^{2}}$$

The analysis was performed for HA and ultrasound-assisted HA treatments. ND was excluded because its environmental conditions varied during natural drying.

### 2.5 Energy consumption

Electrical energy consumption was estimated from the operating power and drying duration of the hot-air and ultrasound systems rather than being directly measured using an electricity meter. For each treatment, the total energy input was calculated as the sum of the estimated energy consumption of the hot-air drying system and the ultrasound system as Eq. (6):

(6) $$E_{total}=E_{HA}+E_{US}$$

where E_total_ is the total energy consumption, kWh; E_HA_ is the energy consumption of the hot-air drying system, kWh; and E_US_ is the energy consumption of the ultrasound system, kWh.

Specific energy consumption (SEC) was then obtained by dividing the total energy input by the mass of water removed as Eq. (7).

(7) $$SEC=\frac{E_{total}}{m_{w}}$$

where SEC is the specific energy consumption, kWh/kg water, and m_w_ is the mass of water removed during drying, kg.

Because the experiments were conducted at a laboratory scale with a sample loading of 120 ± 0.5 g, the calculated SEC values were used primarily for relative comparison among the drying treatments.

The energy-saving rate was calculated by comparing the SEC of each ultrasound-assisted treatment with that of HA as Eq. (8).

(8) $$Energy\;saving\;rate=\frac{S{EC}_{HA}-{SEC}_{i}}{S{EC}_{HA}}\times 100\%$$

where SEC_HA_ is the SEC of the HA group and SEC_i_ is the SEC of each ultrasound-assisted hot-air drying treatment.

### 2.6 Color measurement

Surface color was measured with a CR-410 colorimeter (Konica Minolta, Japan). For each sample, L, a, and b values were recorded after drying and compared with the corresponding fresh-sample values. The total color difference (ΔE) was used to represent the color difference between dried and fresh samples. Lower ΔE values indicate that the dried material retained a color closer to that of the fresh slices. ΔE was calculated using Eq. (9):

(9) $$\Delta E=\sqrt{{\left(L^{*}-L_{0}\right)}^{2}+{\left(a^{*}-a_{0}\right)}^{2}+{\left(b^{*}-b_{0}\right)}^{2}}$$

where L*, a*, and b* represent the color values of the dried samples, while L_0_, a_0_, and b_0_ indicate those of the fresh samples.

### 2.7 Determination of total phenolics (TPC), total flavonoids (TFC), polysaccharide content (PC), and antioxidant capacity

#### 2.7.1 Preparation of sample extract

For spectrophotometric assays, dried slices were milled and screened through a 0.177 mm sieve. Powdered sample, 0.50 g, was extracted with 20 mL of 75% ethanol in a sealed flask. Extraction was performed for 48 h in darkness at room temperature on a shaker operating at 180 r/min. The resulting suspension was centrifuged at 5000 r/min for 10 min under refrigerated conditions at 4 °C. The supernatant was brought to 25 mL with 75% ethanol, stored at 4 °C, and used for TPC, TFC, PC, and DPPH analyses.

#### 2.7.2 Determination of TPC

The TPC was analyzed by a modified Folin–Ciocalteu assay based on the procedure described by Zugazua-Ganado et al. [37]. In each measurement, 20 μL of extract was mixed with 2.0 mL of 10% Folin–Ciocalteu reagent in a test tube. Subsequently, 1.0 mL of 7.5% Na_2_CO_3_ solution was introduced to initiate color development. After vortex mixing for 5 min, the reaction mixture was kept in darkness at 37 °C for 1 h. The absorbance was then recorded at 760 nm using a UV–visible spectrophotometer. Gallic acid was used for calibration, and the results were reported as mg gallic acid equivalent per g dry weight (mg GAE/g DW).

#### 2.7.3 Determination of TFC

The TFC was measured using a modified NaNO_2_-AlCl_3_-NaOH colorimetric method according to Shraim et al. [38]. An aliquot of 100 μL of sample extract was first combined with 2.0 mL of distilled water and 0.3 mL of 5% NaNO_2_ solution. After 5 min of reaction, 0.3 mL of 10% AlCl_3_ solution was added and allowed to react for 1 min. Next, 2.0 mL of 1 mol/L NaOH solution was added to adjust the reaction system to alkaline conditions, followed by thorough mixing. Absorbance was measured at 510 nm using a UV–visible spectrophotometer. Catechin served as the reference compound for the standard curve, and TFC was expressed as mg catechin equivalent per g dry weight (mg CE/g DW).

#### 2.7.4 Determination of PC

PC was determined by the phenol–sulfuric acid colorimetric method with minor modifications following Yue et al. [39]. In each assay, 10 μL of sample extract was mixed with 2.0 mL of 9% phenol solution. After the solution was homogenized, 6.0 mL of concentrated sulfuric acid was rapidly added to the tube. After immediate mixing, the reaction solution was allowed to stand at room temperature for 30 min until full color development occurred. The absorbance was measured at 485 nm using a UV–visible spectrophotometer. Sucrose was selected as the calibration standard, and PC was calculated and expressed as mg/g DW.

#### 2.7.5 Determination of antioxidant capacity

DPPH radical scavenging activity was measured according to a modified method of Baliyan et al. [40]. In brief, 20 μL of extract was combined with methanolic DPPH solution (1 × 10^−4^ mol/L). The mixture was shaken thoroughly and allowed to react for 30 min at room temperature in the dark. Absorbance was subsequently determined at 515 nm using a UV–visible spectrophotometer, with 75% ethanol used as the blank.

DPPH radical scavenging activity was calculated using Eq. (10):

(10) $$DPPH\;scavenging\;activity\left(\%\right)=\frac{\left(A_{0}-A_{s}\right)}{A_{0}}\times 100\%$$

where *A*_0_ is the absorbance of the DPPH control solution without sample extract, and *A*_s_ is the absorbance of the reaction solution containing the sample extract.

### 2.8 Quantification of phenylethanoid and iridoid glycosides by HPLC

The contents of phenylethanoid glycosides and iridoid glycosides in Cistanche samples were determined by high-performance liquid chromatography (HPLC) using a method adapted from our previous study with modifications [16]. The target compounds included rhodioloside, poliumoside, isoacteoside, echinacoside, verbascoside, leonurusoside, and catalpol. Dried Cistanche slices were ground and passed through a 0.177 mm sieve. A 1.00 g portion of sample powder was accurately weighed and mixed with 50 mL of 50% methanol in an amber bottle. After standing for 30 min, the bottle was weighed and subjected to ultrasonic extraction at 250 W and 40 kHz for 40 min. After extraction, any mass loss was compensated with 50% methanol. The extract was thoroughly mixed, filtered, and passed through a 0.22 μm membrane before HPLC analysis.

HPLC analysis was performed using an Agilent 1100 high-performance liquid chromatograph (Agilent Technologies, USA) equipped with an Agilent Eclipse XDB-C18 column (250 mm × 4.6 mm, 5 μm). The column temperature was maintained at 30 °C and the flow rate was 1.0 mL/min. The mobile phase consisted of water (A) and acetonitrile (B), with the following gradient program: 0–6 min, 0–20% B; 6–10 min, 20–25% B; 10–15 min, 25–30% B; 15–20 min, 30–35% B; 20–24 min, 35–65% B; 24–26 min, 65–45% B; and 26–28 min, 45–10% B. Detection was performed at 205 nm. Each sample was analyzed in triplicate.

### 2.9 Scanning electron microscopy (SEM)

Dried samples from each treatment were sectioned into approximately 2 mm × 2 mm pieces for SEM observation. Each piece was mounted on an aluminum stub with conductive adhesive and coated with a thin gold layer for 90 s before imaging. Micrographs were obtained with an S-3400N scanning electron microscope (Hitachi, Japan) at 5.0 kV and 300× magnification. Representative areas were selected to compare tissue collapse, pore formation, and channel connectivity among treatments.

### 2.10 Statistical analysis

Each drying treatment was independently performed three times using separately prepared batches of 120 ± 0.5 g Cistanche slices. Each independent drying run constituted one experimental replicate. Unless otherwise stated, the results are expressed as mean ± standard deviation of the three independent replicates. Statistical comparisons among treatments were performed by one-way ANOVA followed by Duncan’s multiple range test at p < 0.05. Different lowercase letters in the figures indicate significant differences among treatments. Figures were prepared in OriginPro 2021, and statistical analyses were completed using SPSS 26.0.

## 3 Results and discussion

### 3.1 Drying characteristics and effective moisture diffusivity (*D_eff_*)

As shown in Fig. 2, the MR of all samples decreased continuously with drying time, although the rate of decrease varied among treatments. The HA group required 480 min to reach the drying endpoint, whereas SFU1, SFU2, and SFU3 shortened the time to 420, 390, and 330 min, corresponding to reductions of 12.50%, 18.75%, and 31.25%, respectively, compared with HA. DFU and TFU further enhanced the drying process. DFU1, DFU2, and TFU reached the drying endpoint within 270 min, corresponding to a 43.75% reduction relative to HA, while DFU3 required 300 min, corresponding to a 37.50% reduction. These results indicate that ultrasound assistance effectively intensified the dehydration of Cistanche slices and that DFU and TFU generally outperformed SFU. The accelerated drying process was likely associated with ultrasound-induced cavitation, mechanical vibration, and microstreaming, which disrupted the surface boundary layer and facilitated moisture transfer from the material surface. Meanwhile, periodic compression and expansion of the cellular tissue promote the formation of pores, cracks, and capillary channels, allowing internal moisture to migrate more readily toward the surface. A study on ultrasound-strengthened air-impingement drying of Chaenomeles sinensis slices similarly showed that ultrasound disrupted cell walls, loosened tissue structure, increased pores and microchannels, and reduced the total drying time by 12.50–43.75% [18]. It should be noted that the intensification effect of different frequency combinations did not increase linearly with the number of frequencies. Because single-frequency ultrasound has a fixed frequency, its acoustic action range and cavitation response may be relatively limited, and local regions may experience acoustic energy concentration or attenuation, resulting in uneven mechanical disturbance of the material. By contrast, dual- or triple-frequency ultrasound can stimulate a broader range of bubble responses and more complex microstreaming through frequency superposition, thereby expanding the effective cavitation region and mass-transfer channels [25].

**Fig. 2:**
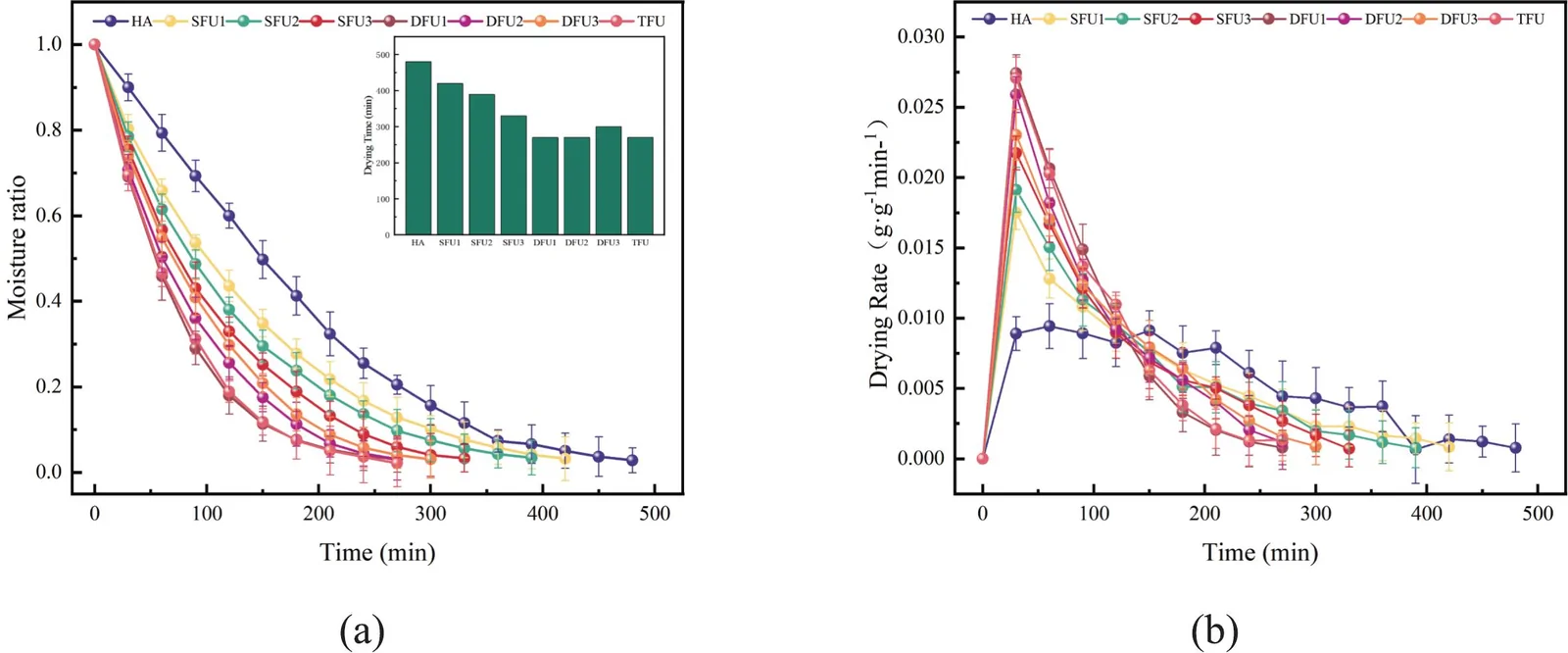
Drying characteristics of Cistanche slices under different drying treatments (a) moisture ratio curves and (b) drying rate curves.

However, DFU1, DFU2, and TFU all reached the shorter endpoint time of 270 min, whereas DFU3 required 300 min, indicating differences in frequency matching among dual-frequency combinations. The DFU1 combination may have produced stronger tissue disturbance through the combined action of two low-frequency acoustic fields, resulting in a pronounced early-stage moisture loss rate. The TFU group simultaneously introduced three frequencies and may have enhanced the generation frequency and distribution of cavitation bubbles through composite frequency resonance, forming wider and intersecting microstreaming channels within the cellular matrix. This could increase the probability of cell wall rupture and intercellular water permeation, thereby promoting rapid internal moisture migration to the surface. Xu et al. reported that DFU shortened the vacuum freeze-drying time of strawberry slices more effectively than single-frequency ultrasound and that frequency mode affected final quality [26]. A study on pineapple slices also showed that TFU was advantageous for shortening drying time and improving quality [27].

To further quantify moisture transport during drying, the *D_eff_* was estimated from the experimental MR data. As shown in Table 2, the *D_eff_* of HA was 3.36 × 10^−10^ m^2^/s, whereas the ultrasound-assisted treatments showed values ranging from 3.49 × 10^−10^ to 6.11 × 10^−10^ m^2^/s. Among the SFU treatments, *D_eff_* increased with frequency and reached 4.52 × 10^−10^ m^2^/s for SFU3. Higher values were obtained for the DFU treatments, ranging from 5.17 × 10^−10^ to 5.64 × 10^−10^ m^2^/s. TFU exhibited the highest *D_eff_* of 6.11 × 10^−10^ m^2^/s, approximately 81.7% higher than that of HA. The coefficients of determination ranged from 0.9858 to 0.9984, indicating good agreement with the linearized diffusion model.

**Table 2:** Effective moisture diffusivity of Cistanche slices under different drying treatments.

Treatment	*D_eff_* (×10^−10^ m^2^/s)	*R^2^*
HA	3.363	0.9858
SFU1	3.495	0.9959
SFU2	3.735	0.9984
SFU3	4.523	0.9941
DFU1	5.643	0.9917
DFU2	5.618	0.9956
DFU3	5.171	0.9961
TFU	6.112	0.9984

### 3.2 Energy consumption

To evaluate whether ultrasound-assisted drying could offset the additional energy input from the ultrasound system while improving drying efficiency, the SEC and energy saving rate relative to HA were calculated. The variations in SEC and energy-saving rate among the drying treatments are presented in Fig. 3. The SEC of HA was 112.44 kWh/kg water. The SEC values of SFU1, SFU2, and SFU3 decreased to 109.12, 101.84, and 85.49 kWh/kg water, respectively, corresponding to energy saving rates of 2.95%, 9.43%, and 23.97%. These results indicate that although SFU introduced additional energy input, the reduction in hot-air energy consumption resulting from shorter drying time was sufficient to decrease the unit energy consumption for water removal. Tao et al. also found that both airborne and contact ultrasound improved drying efficiency and reduced energy consumption during blackberry air drying, and that acoustic energy coupling mode significantly influenced energy efficiency [41]. DFU showed more pronounced energy-saving performance. The SEC values of DFU1, DFU2, and DFU3 were 76.42, 76.15, and 84.73 kWh/kg water, respectively, corresponding to energy saving rates of 32.03%, 32.28%, and 24.65% relative to HA. Among them, DFU2 exhibited the lowest SEC and highest energy saving rate, suggesting that the 25 + 40 kHz combination achieved a favorable balance between drying acceleration and energy input under the present experimental conditions. The SEC of TFU was 81.16 kWh/kg water, corresponding to a 27.82% reduction relative to HA, but remained higher than those of DFU1 and DFU2. Since the total ultrasonic power was fixed at 120 W for all ultrasound treatments, the differences in SEC primarily reflected differences in drying performance under the tested frequency modes rather than differences in nominal ultrasonic power. Similarly, intermittent MFU enhanced vacuum far-infrared drying of rhubarb indicated that continuous MFU could promote moisture migration but might also increase energy consumption and structural damage, whereas intermittent regulation helped balance energy efficiency and quality [42]. A study on MFU-assisted vacuum far-infrared drying of Cornus officinalis also showed that TFU significantly improved quality, although its energy consumption could be slightly higher than that of some control or single-frequency treatments [43].

**Fig. 3:**
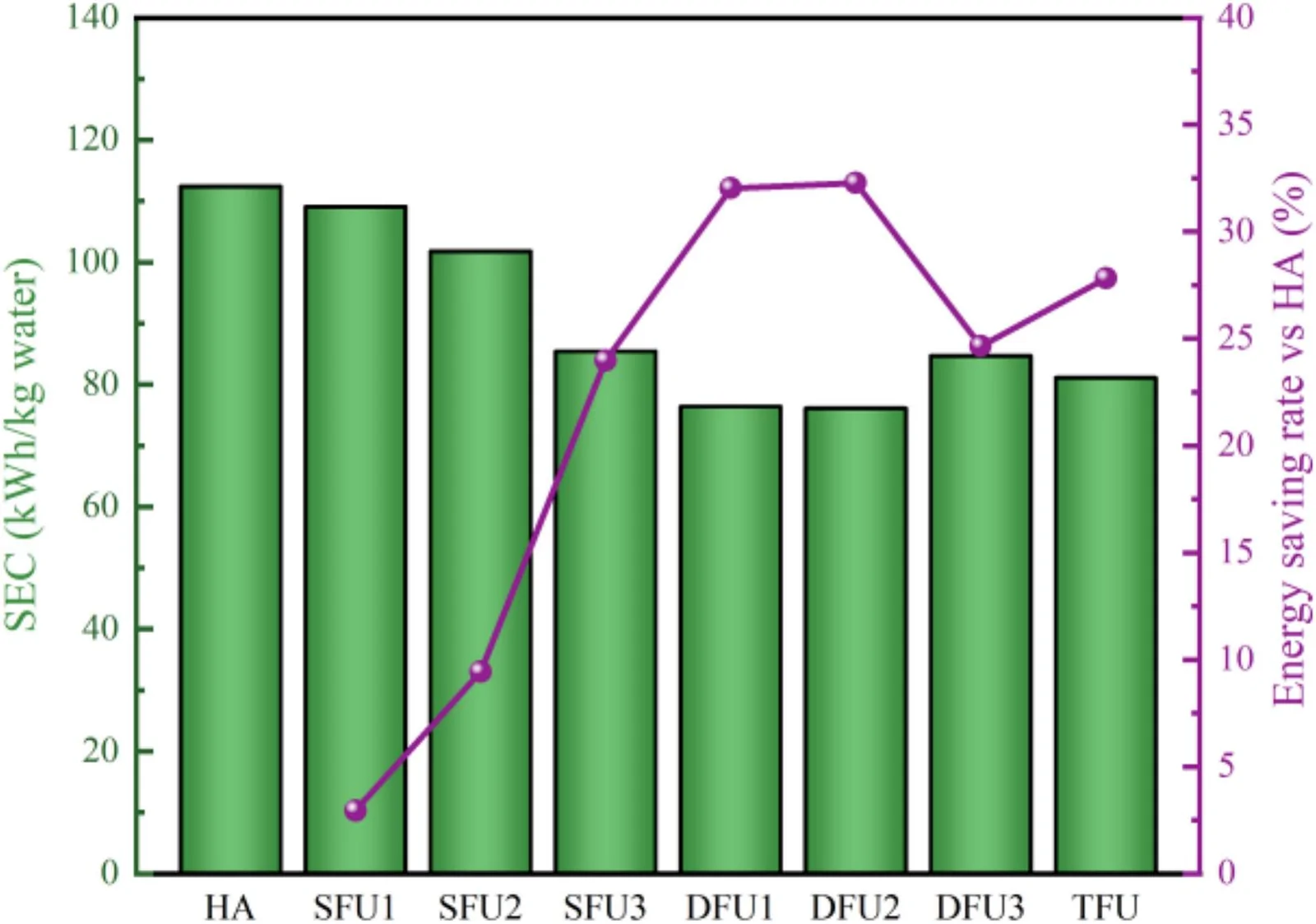
Effects of different drying treatments on the specific energy consumption and energy-saving rate of Cistanche slices.

### 3.3 Color

The visual appearance of dried Cistanche slices was evaluated using color parameters, and the corresponding L, a, b, and ΔE values for each treatment are summarized in Fig. 4. In the ND group, L increased to 75.80, whereas b decreased to 14.24, indicating that natural sun drying may have reduced yellowness and caused hue deviation. In the HA group, L decreased to 57.43 and ΔE reached 12.36, demonstrating substantial color deterioration caused by hot-air drying alone. Prolonged thermal exposure during HA may promote non-enzymatic browning, phenolic oxidation, sugar degradation, and tissue shrinkage, thereby reducing lightness and increasing total color difference. Ai et al. reported that color changes in Cistanche processing were jointly associated with phenylethanoid glycosides, sugars, polysaccharides, microstructure, and starch gelatinization, indicating that color deterioration is not caused by thermal effects alone but by the combined effects of tissue structure and compositional transformation [5]. Compared with HA, ultrasound-assisted treatments generally improved color retention. Among the dried samples, TFU preserved the lightness most effectively, with an L* value of 66.33, and showed the smallest color deviation from the fresh material, as indicated by a ΔE value of 6.31. DFU1 also showed good color retention, with a ΔE value of 6.36, close to that of TFU. This improvement can be explained by the fact that ultrasound shortened drying time and greatly reduced the exposure of the material to high-temperature hot air, thereby slowing browning reactions. In addition, ultrasound-induced pore formation promoted internal moisture migration and alleviated surface hardening and local overheating. Faster dehydration may also reduce the duration of enzymatic and oxidative reactions. Wu et al. also linked the quality improvement of ultrasound-assisted vacuum far-infrared dried Cistanche slices to structural modification, reporting better color preservation and rehydration performance after ultrasound treatment [16]. However, the a value of SFU3 increased to 4.93, indicating that stronger or uneven ultrasound action may also promote reddish-brown discoloration.

**Fig. 4:**
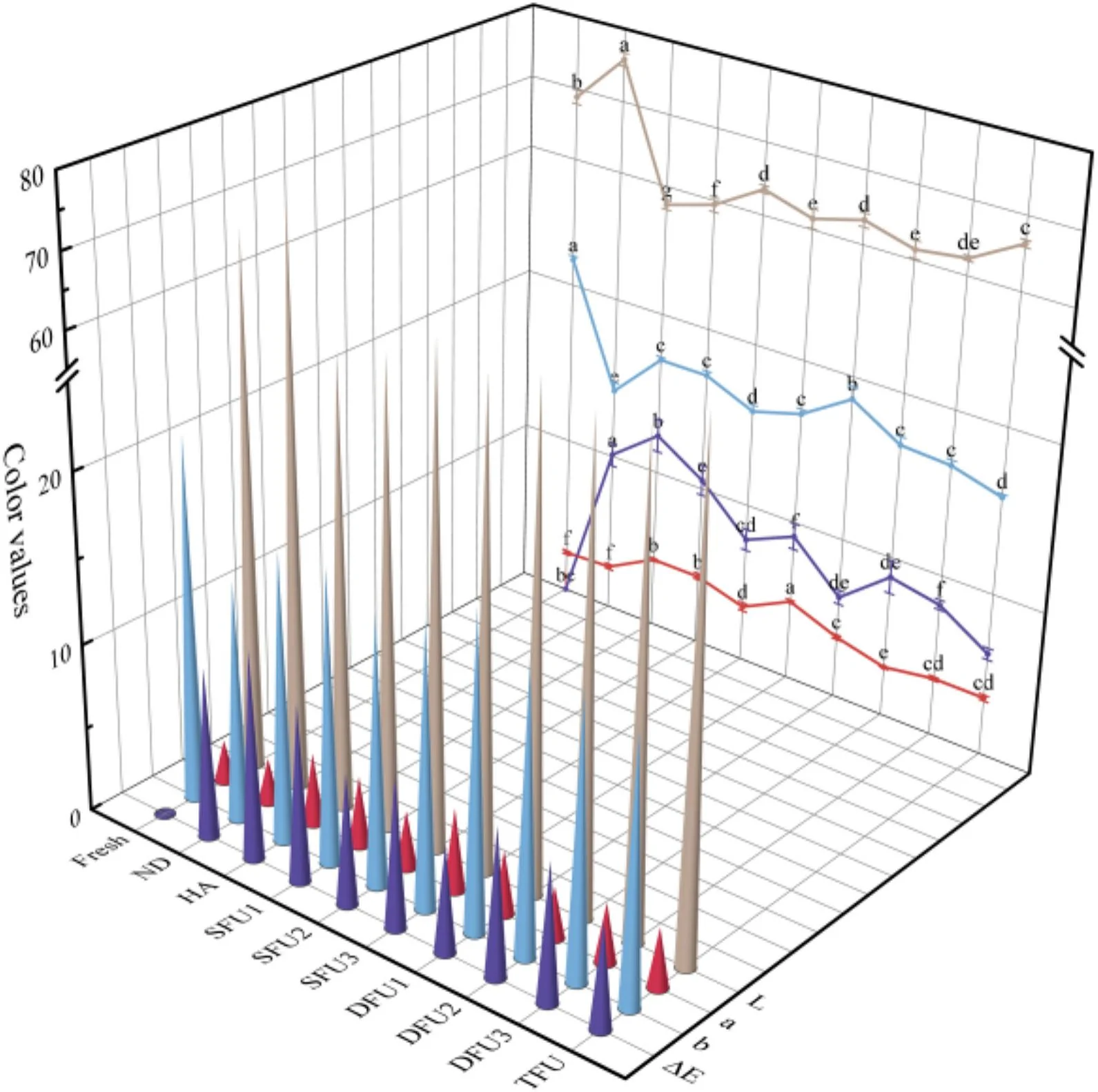
Variations in L, a, b, and ΔE values of Cistanche slices after different drying treatments.

### 3.4 TPC

The variation in TPC among Cistanche slices subjected to different drying treatments is presented in Fig. 5. Different drying treatments clearly affected the TPC. The ND and HA groups showed relatively low values of 20.17 and 21.66 mg GAE/g DW, respectively. The values for SFU1, SFU2, and SFU3 were 23.22, 25.99, and 31.03 mg GAE/g DW, representing increases of 7.17%, 19.99%, and 43.24%, respectively, compared with HA. DFU1, DFU2, and DFU3 reached 32.87, 35.98, and 38.97 mg GAE/g DW, respectively, whereas TFU showed the highest value of 52.60 mg GAE/g DW. Some phenolic compounds in Cistanche may be bound to cell-wall polysaccharides, proteins, or other matrix components. Tissue shrinkage and cell-wall collapse caused by conventional HA may restrict solvent diffusion during extraction, leading to a lower measured phenolic content. In contrast, ultrasonic cavitation and mechanical disturbance can disrupt cell-wall structures, increase pores and cracks, and promote the release of embedded or bound phenolics. In a study on MFU extraction from purple eggplant peels, TFU significantly increased the extraction yields of total phenolics and anthocyanins compared with SFU and DFU, indicating that multi-frequency acoustic fields favor the release of phenolic compounds from plant matrices [44]. MFU-assisted enzymatic extraction of coconut by-products also demonstrated that TFU more effectively disrupted cell structures and increased extraction yield, total phenolic content, and DPPH radical scavenging activity [45]. Moreover, ultrasound-assisted drying shortened drying time, which may reduce the risks of phenolic oxidation and thermal degradation. Therefore, the higher measured TPC in the TFU group may reflect a combination of reduced degradation during drying and enhanced extractability resulting from ultrasound-induced structural disruption.

**Fig. 5:**
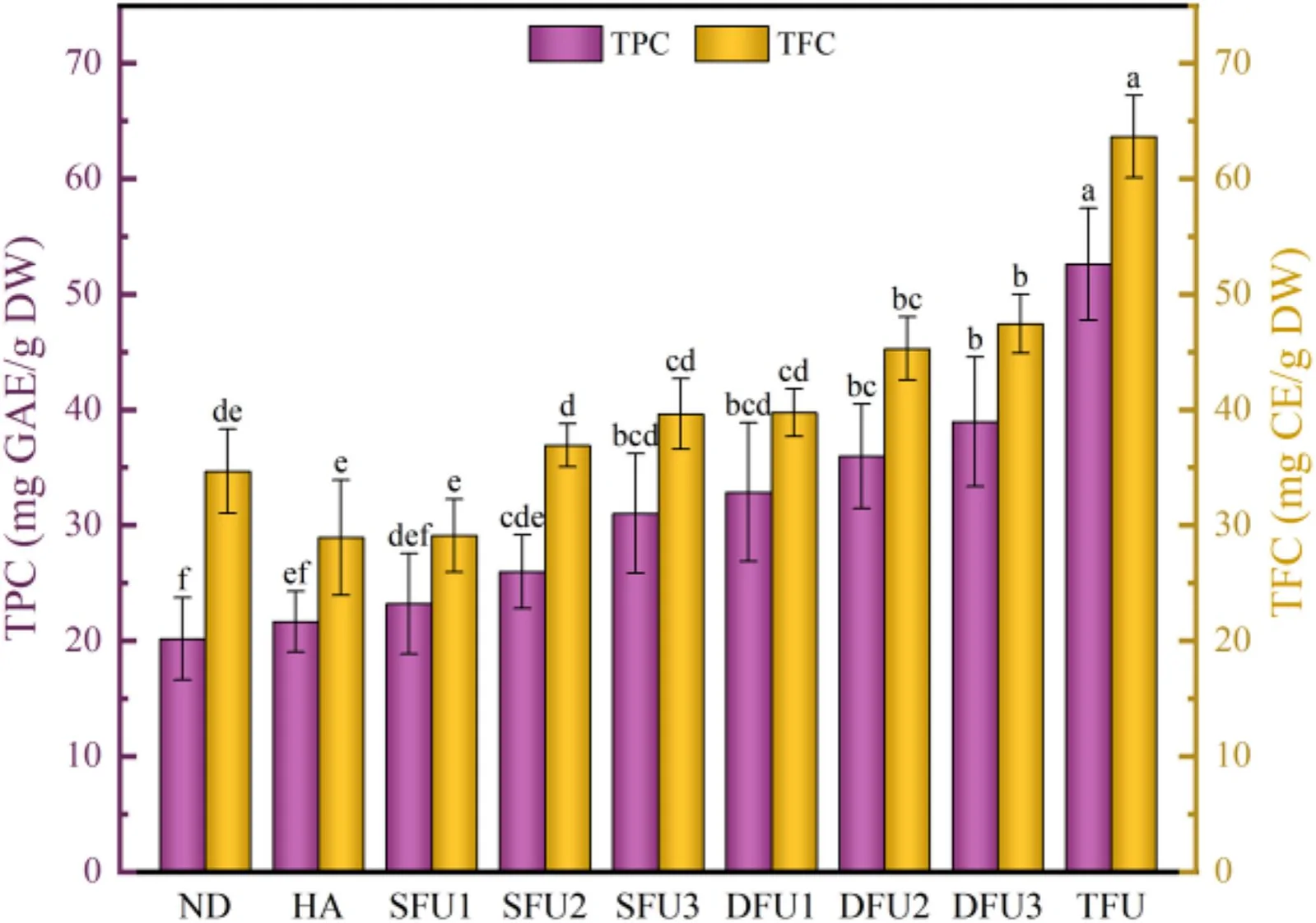
TPC and TFC of Cistanche slices under different drying treatments.

### 3.5 TFC

Changes in TFC across the different drying treatments are also summarized in Fig. 5. The trend in TFC was similar to, but not identical with, that of TPC. The TFC measured in the HA treatment was 28.94 mg CE/g DW, which was lower than the value obtained for ND (34.67 mg CE/g DW), suggesting that HA alone may have promoted flavonoid degradation or reduced flavonoid extractability. SFU1 was close to HA, with a value of 29.12 mg CE/g DW, whereas SFU2 and SFU3 increased to 36.97 and 39.63 mg CE/g DW, respectively. DFU1, DFU2, and DFU3 reached 39.75, 45.30, and 47.44 mg CE/g DW, respectively, while TFU exhibited the highest value of 63.66 mg CE/g DW. Ultrasound can loosen cell walls through cavitation, shear, and microstreaming effects, thereby improving the release efficiency of flavonoids from vacuoles and cytoplasm. Shorter drying time also reduces flavonoid oxidation and thermal degradation in the hot-air environment [15]. Notably, DFU1 and TFU had the same drying endpoint time, but the TFC of DFU1 was markedly lower than that of TFU, suggesting that the measured TFC was not determined solely by thermal exposure time, but may also have been influenced by differences in tissue disruption and flavonoid extractability induced by the different frequency combinations. DFU1 may have generated stronger low-frequency mechanical disturbance, which was beneficial for moisture migration but may also have increased flavonoid exposure to oxidative environments. TFU may have produced a more uniform porous structure, allowing a better balance between mass-transfer enhancement and structural protection. Studies on MFU-improved cherry drying have similarly shown that MFU treatment is often associated with higher TPC, TFC, and antioxidant capacity [46]. In sea buckthorn juice fermentation, MFU was also shown to enhance functional quality by modifying microstructure and improving the accessibility of bioactive compounds [47]. Accordingly, the higher TFC measured after ultrasound-assisted drying should be interpreted as the combined result of possible preservation during drying and enhanced extraction from the modified tissue matrix, rather than solely as improved flavonoid retention.

### 3.6 PC

Polysaccharides are important functional components of Cistanche. The polysaccharide content of Cistanche slices after different drying treatments is compared in Fig. 6. The PC value after HA treatment was 209.56 mg/g DW, which was lower than that of ND (223.13 mg/g DW), implying that HA alone may have reduced polysaccharide retention or limited its subsequent extraction. The values for SFU1, SFU2, and SFU3 were 237.93, 344.72, and 306.89 mg/g DW, respectively, with SFU2 showing a favorable effect on polysaccharide release or retention. Among the dual-frequency treatments, DFU1, DFU2, and DFU3 showed values of 218.47, 263.29, and 323.89 mg/g DW, respectively, while TFU showed the highest value of 370.63 mg/g DW. The change in PC was not fully synchronized with drying time. DFU1 and TFU both reached the drying endpoint within 270 min, but the PC of DFU1 was only 218.47 mg/g DW, whereas TFU reached 370.63 mg/g DW. This indicates that polysaccharide variation was influenced not only by thermal exposure time but also by the pattern of cell-wall disruption, pore connectivity, solvent accessibility, and molecular stability of polysaccharides. Appropriate ultrasound may disrupt cell walls and the middle lamella, thereby improving polysaccharide release and extraction efficiency. However, excessive mechanical action may cause polysaccharide chain scission, degradation, or oxidation, reducing the measured content or altering structural characteristics [48]. Studies on MFU in lotus root starch/xanthan gum-stabilized emulsions and rose polyphenol-modified starch have shown that ultrasound can alter particle size, surface morphology, and intermolecular interactions in polysaccharide or starch-based matrices, suggesting that the content and functional properties of polysaccharide-like materials may be influenced by both structural changes and extraction accessibility [32], [33]. Studies on Cistanche polysaccharides also indicate that polysaccharide extraction, structure, and activity are highly dependent on processing and extraction conditions [48]. Therefore, the higher measured PC in the TFU group may have resulted from both differences in polysaccharide stability during drying and improved extraction accessibility associated with tissue opening.

**Fig. 6:**
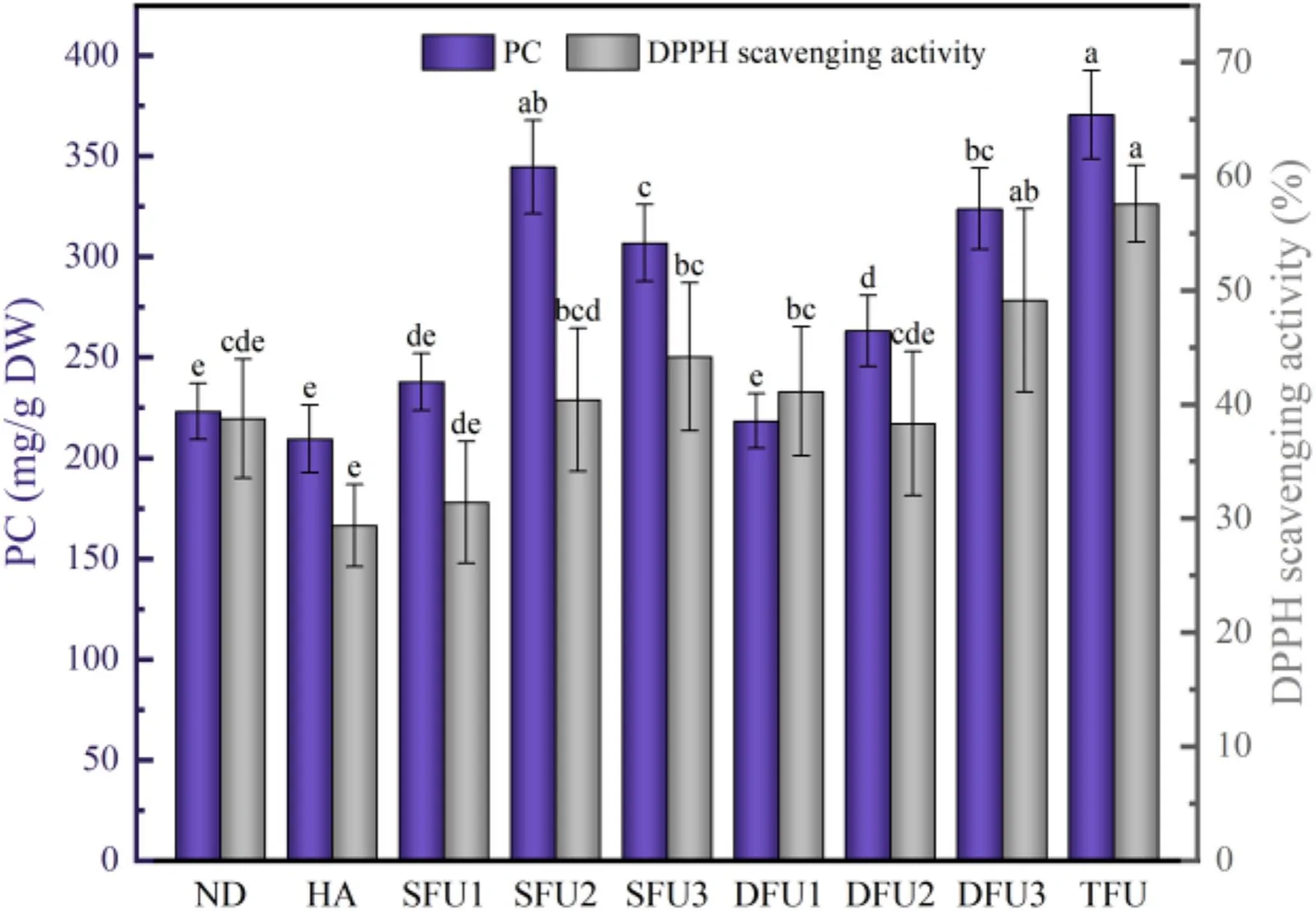
PC and DPPH radical scavenging activity of Cistanche slices under different drying treatments.

### 3.7 Antioxidant property

Fig. 6. further presents the DPPH radical scavenging activity of Cistanche slices obtained under the different drying treatments. Different treatments markedly affected antioxidant activity. The DPPH inhibition rate of the HA group was 29.39%, the lowest among all treatments, whereas that of the ND group was 38.76%. The values for SFU1, SFU2, and SFU3 were 31.42%, 40.42%, and 44.18%, respectively, showing an increasing trend with changes in single-frequency ultrasound. Among the dual-frequency treatments, DFU1, DFU2, and DFU3 showed values of 41.14%, 38.32%, and 49.13%, respectively, with DFU3 performing best; TFU showed the highest value of 57.59%. Overall, the antioxidant activity was broadly consistent with the changes in total phenolics, total flavonoids, and polysaccharides. The TFU group simultaneously showed the highest contents of total phenolics, total flavonoids, and polysaccharides, as well as the highest antioxidant capacity, suggesting that the antioxidant activity of dried Cistanche samples may be jointly contributed by phenolics, flavonoids, polysaccharides, phenylethanoid glycosides, and other compounds. Variable-frequency ultrasound-assisted hot-air drying of cherry similarly improved TPC, antioxidant activity, and overall quality while shortening drying time [35]. However, although DFU2 had relatively high total phenolic and total flavonoid contents, its DPPH inhibition rate was lower than those of SFU3, DFU1, and DFU3. This suggests that antioxidant activity was not determined solely by total-content indicators, but also depended on molecular structure, the number of phenolic hydroxyl groups, glycosylation degree, spatial conformation, and synergistic interactions among components. Studies on multi-frequency ultrasound processing of sea buckthorn and related plant systems also suggest that microstructural changes and improved extractability of bioactive compounds can significantly affect antioxidant performance [47].

### 3.8 Phenylethanoid glycosides

Phenylethanoid glycosides are among the most representative characteristic bioactive compounds in Cistanche and are important indicators of functional quality and medicinal value. Echinacoside and verbascoside are generally considered key quality-control markers for Cistanche, whereas rhodioloside, poliumoside, and isoacteoside collectively reflect the composition and processing stability of phenylethanoid glycosides [49]. The contents of phenylethanoid glycosides in Cistanche slices under different drying conditions are shown in Fig. 7. Different drying treatments significantly affected the five phenylethanoid glycoside monomers. The TFU group showed the highest rhodioloside content (0.2585 mg/g), which was significantly higher than that of the other treatments. ND and SFU2 were at relatively high levels. Isoacteoside and echinacoside also reached their highest levels in the TFU group, indicating that TFU was favorable for the retention or release of most phenylethanoid glycosides. The change in poliumoside showed a clear dependence on frequency combination. ND, SFU2, and DFU3 showed higher levels, whereas SFU3, DFU1, and DFU2 showed significantly lower levels. Verbascoside was highest in ND. Although TFU was significantly higher than HA and the other ultrasound treatments, it was still lower than ND, suggesting that verbascoside may be more sensitive to hot-air drying conditions. Huang et al. reported that the degradation of echinacoside and verbascoside during drying of Cistanche was related to both enzymatic reactions and thermal degradation, and that pretreatment method significantly affected the degradation pathways of phenylethanoid glycosides [49]. Ultrasound shortened drying time and thereby reduced exposure to hot air while promoting compound release. However, excessive or poorly matched structural disruption may increase the contact of compounds with oxygen, heat, and residual enzymes, accelerating the degradation of certain monomers. Overall, TFU was beneficial for rhodioloside, isoacteoside, echinacoside, and verbascoside, but it was not optimal for poliumoside. This indicates that TFU did not exert a uniform promoting effect on all phenylethanoid glycoside monomers. Differences in cellular localization, binding state, thermal stability, and degradation pathway may cause different monomers to respond differently to frequency combinations.

**Fig. 7:**
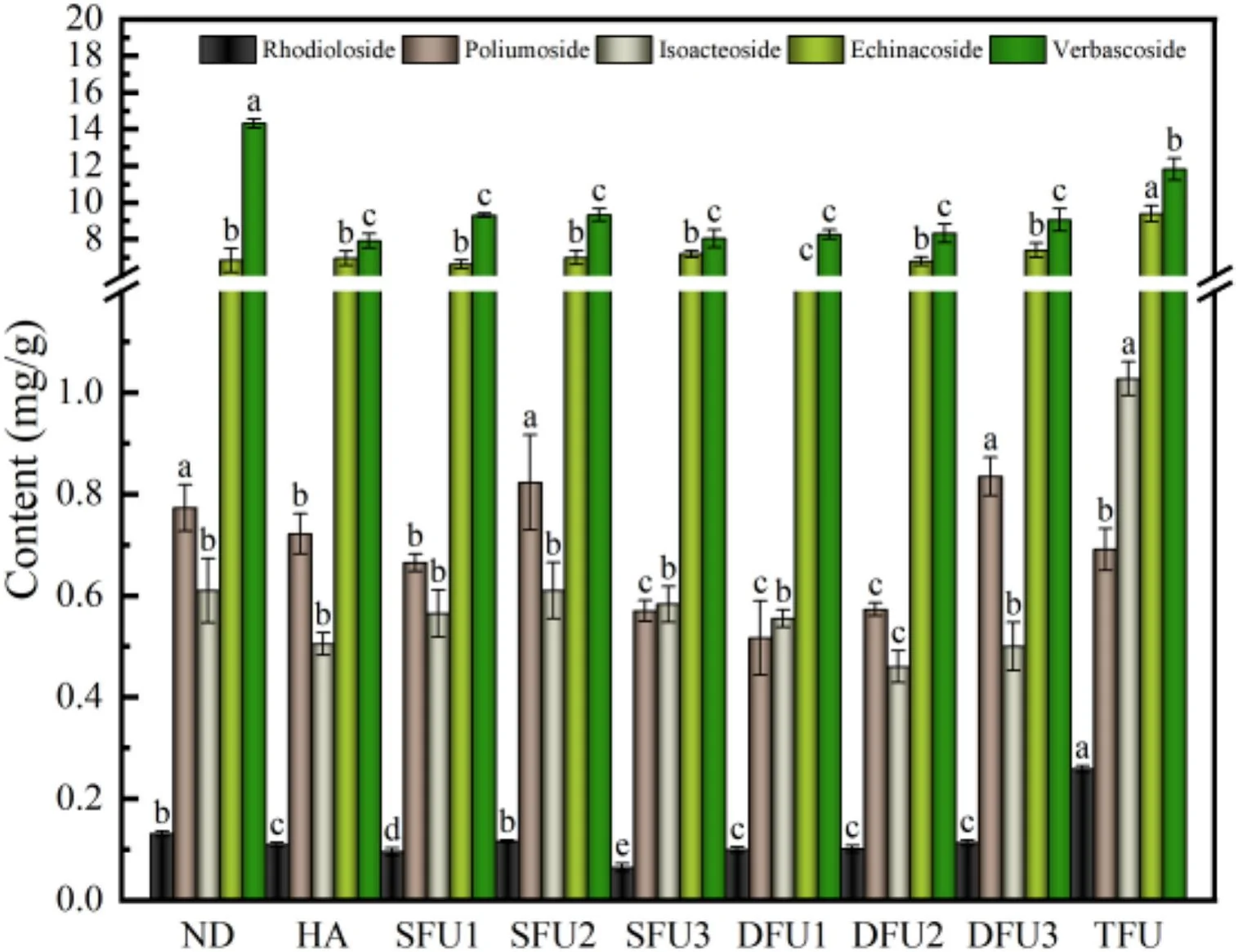
Variations in individual phenylethanoid glycosides of Cistanche slices subjected to different drying treatments.

### 3.9 Iridoid glycosides

Iridoid glycosides represent another important class of bioactive compounds in Cistanche and, together with phenylethanoid glycosides and polysaccharides, contribute to the quality basis of Cistanche [1], [3]. Fig. 8 shows that leonurusoside levels were highest in the ND and TFU treatments, reaching 0.35577 and 0.38891 mg/g, respectively. Compared with HA, TFU increased leonurusoside by approximately 38.6%. By contrast, SFU1 showed a lower value than HA, indicating that single low-frequency ultrasound may have caused local structural damage or may not have favored the stability of this compound. The trend for catalpol differed from that of leonurusoside. SFU1, DFU3, and TFU were all at the highest significance level, with values of 0.38699, 0.39292, and 0.40744 mg/g, respectively. DFU1 showed the lowest value of 0.28698 mg/g. A possible explanation is that some low-frequency combinations may cause strong structural disruption, exposing compounds more readily to heat, oxygen, or enzymatic environments and thereby promoting degradation. Compared with HA, TFU increased catalpol by approximately 32.7%. These results indicate that TFU generally favored the retention or release of iridoid glycosides, but the response of catalpol to frequency combination was not entirely dependent on triple-frequency ultrasound. Iridoid glycosides may be more susceptible to hydrolysis, thermal degradation, and enzymatic transformation than to antioxidant reactions alone [16]. In addition, SFU1 and DFU3 also exhibited high catalpol levels, suggesting that catalpol responded differently to ultrasonic frequency combinations than leonurusoside and did not rely solely on triple-frequency ultrasound. The lower catalpol content in DFU1, despite its rapid drying, further indicates that strong mass-transfer enhancement does not necessarily correspond to optimal retention of bioactive compounds.

**Fig. 8:**
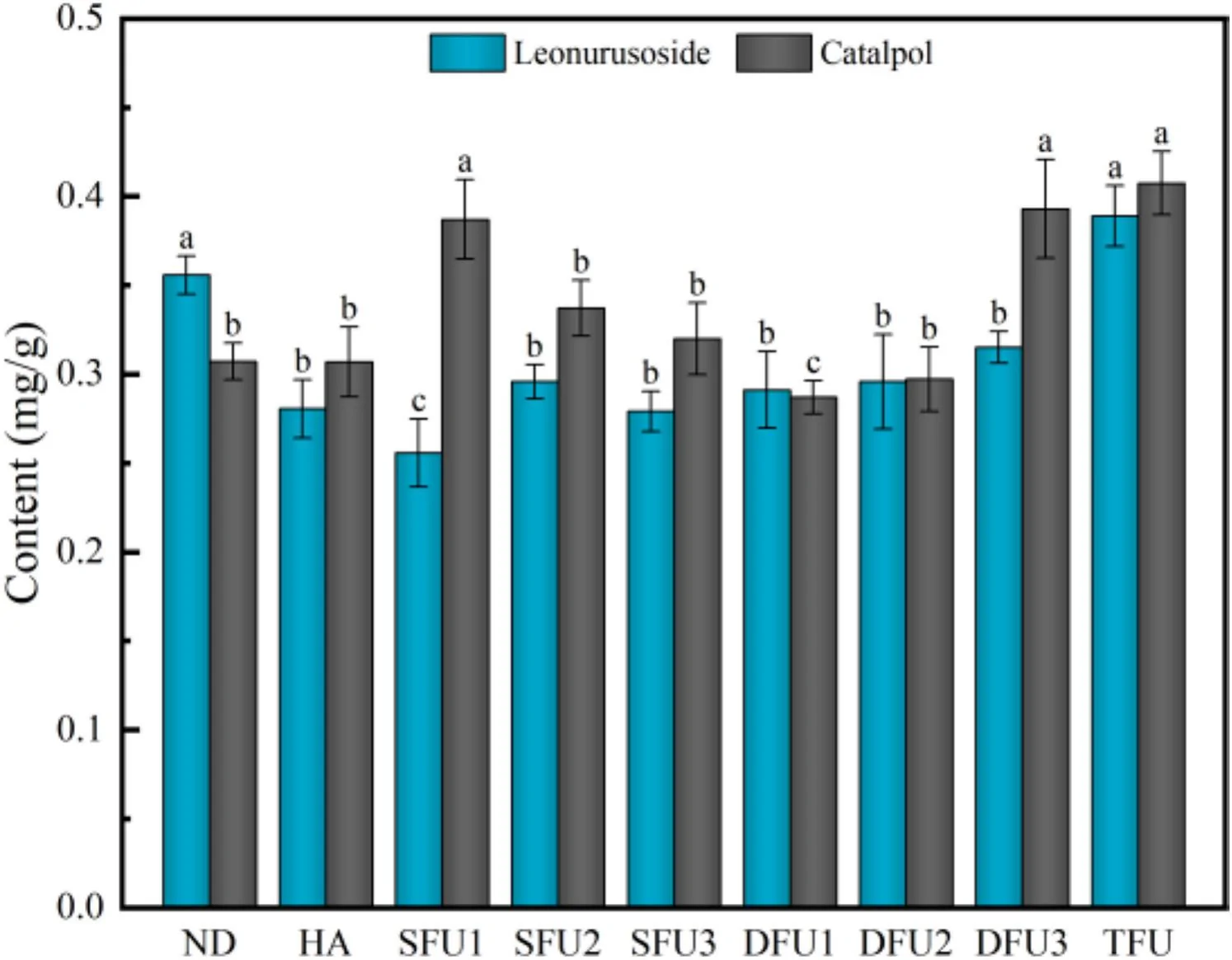
Contents of iridoid glycosides in Cistanche slices under different drying treatments.

### 3.10 Microstructure

SEM images (Fig. 9) showed that different drying methods substantially altered the microstructure of Cistanche slices. ND samples were relatively compact overall, with local wrinkling and smooth collapse and without obvious pores, indicating that slow moisture migration during natural sun drying may have gradually compacted the tissue. The HA group exhibited pronounced tissue collapse, surface shrinkage, and local fragmentation, with fewer pore channels. This corresponded to its longer drying time, higher ΔE, and lower retention of bioactive compounds. Prolonged hot-air treatment can easily cause surface hardening and cell-wall collapse, restricting further internal moisture migration during the later drying stage and increasing the thermal-oxidative exposure of active compounds. Ultrasound treatments markedly changed the degree of tissue opening. After SFU treatment, SFU1 showed some cracks and rough structures, but the channels remained insufficient. SFU2 showed larger pores and layered channels, whereas SFU3 exhibited more cracks and small pores. DFU treatments further enhanced porosity. DFU2 and DFU3 displayed more dispersed pores and rough surfaces. However, DFU1 showed local structural destruction with large cracks and pores, which may explain the lower polysaccharide and selected bioactive compound contents. In contrast, the TFU group exhibited a more loosened, porous, and lamellar structure with more open internal channels. This structure may have reduced internal moisture diffusion resistance while avoiding excessive collapse and local thermal damage, consistent with its shorter drying time and higher TPC, TFC, PC, and antioxidant capacity. The MFUS-VFIR study on Cornus officinalis also showed that multi-frequency ultrasound formed honeycomb-like pores and larger pore diameters, expanded microchannels, and reduced mass-transfer resistance [43]. In addition, related studies have shown that multi-frequency ultrasound can influence quality formation by modifying microstructure [50].

**Fig. 9:**
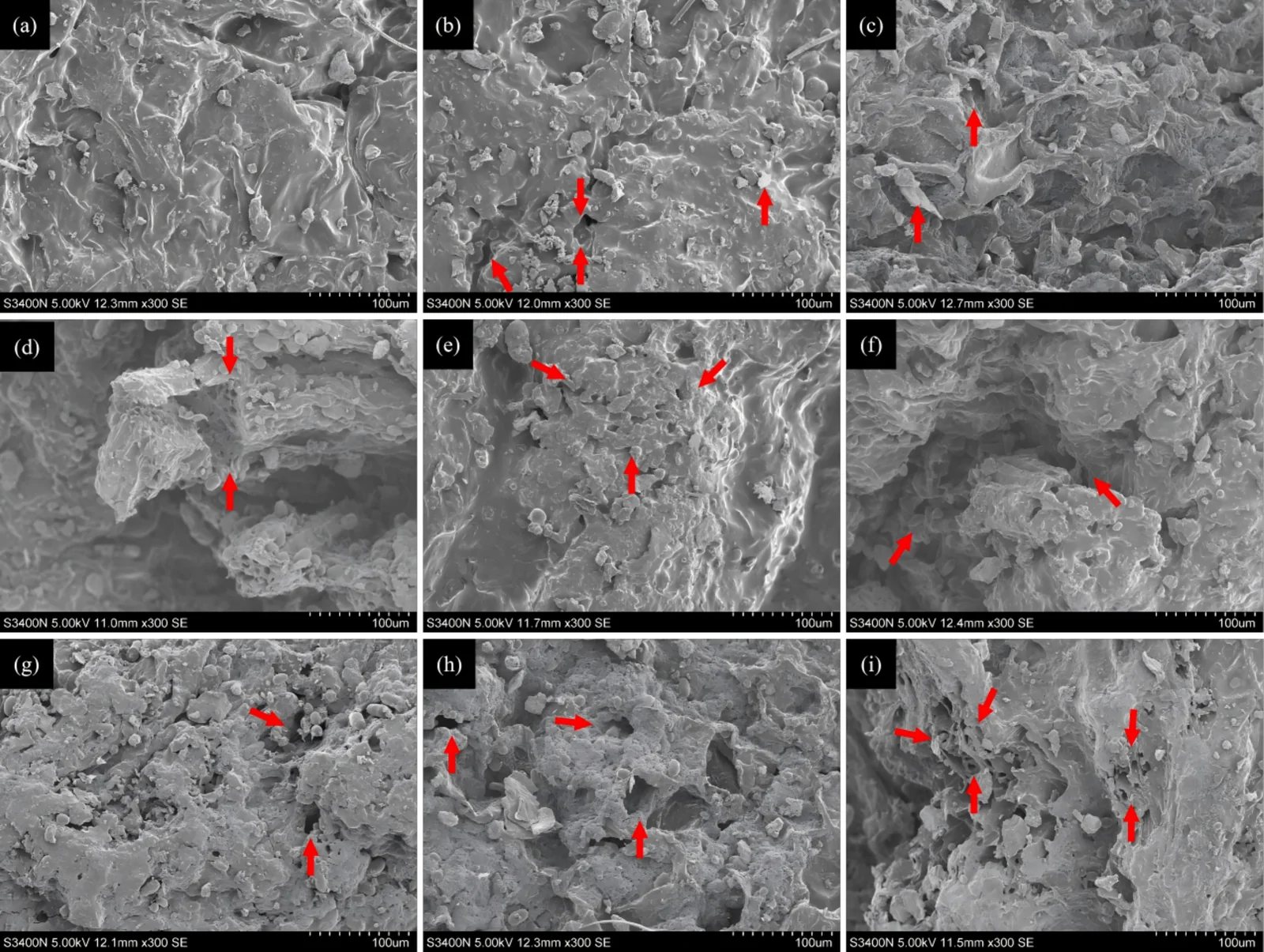
Microstructural morphology of Cistanche slices subjected to different drying treatments (a) ND, (b) HA, (c) SFU1, (d) SFU2, (e) SFU3, (f) DFU1, (g) DFU2, (h) DFU3, and (i) TFU.

### 3.10 Hierarchical clustering analysis

To visually demonstrate the overall similarity among different drying treatments in terms of quality, energy consumption, and bioactive compound indicators, hierarchical clustering analysis was performed based on standardized indicators. In the heatmap (Fig. 10), red indicates relatively high standardized values and blue indicates relatively low standardized values. The clustering results showed that TFU was clearly separated from the other treatments and exhibited relatively high values for most bioactive and functional-quality indicators. This indicates that TFU generated a quality profile distinct from that of the other treatments, mainly characterized by higher measured levels of multiple bioactive compounds. HA and SFU1 were located close to each other in the clustering map, suggesting that 25 kHz single-frequency ultrasound produced only limited overall quality improvement compared with hot-air drying alone. This was consistent with the relatively small improvements observed for total phenolic content, total flavonoid content, antioxidant capacity, and SEC in SFU1. The locations of SFU2 and SFU3 indicated that changes in single-frequency ultrasound improved sample quality to some extent, although the overall effect remained weaker than that of combined-frequency ultrasound treatments. DFU1 and DFU2 exhibited lower SEC and higher energy saving rates, but their bioactive compound retention was not consistently superior to that of TFU. DFU3 showed better performance in poliumoside content and antioxidant capacity, indicating that different dual-frequency combinations produced different effects.

**Fig. 10:**
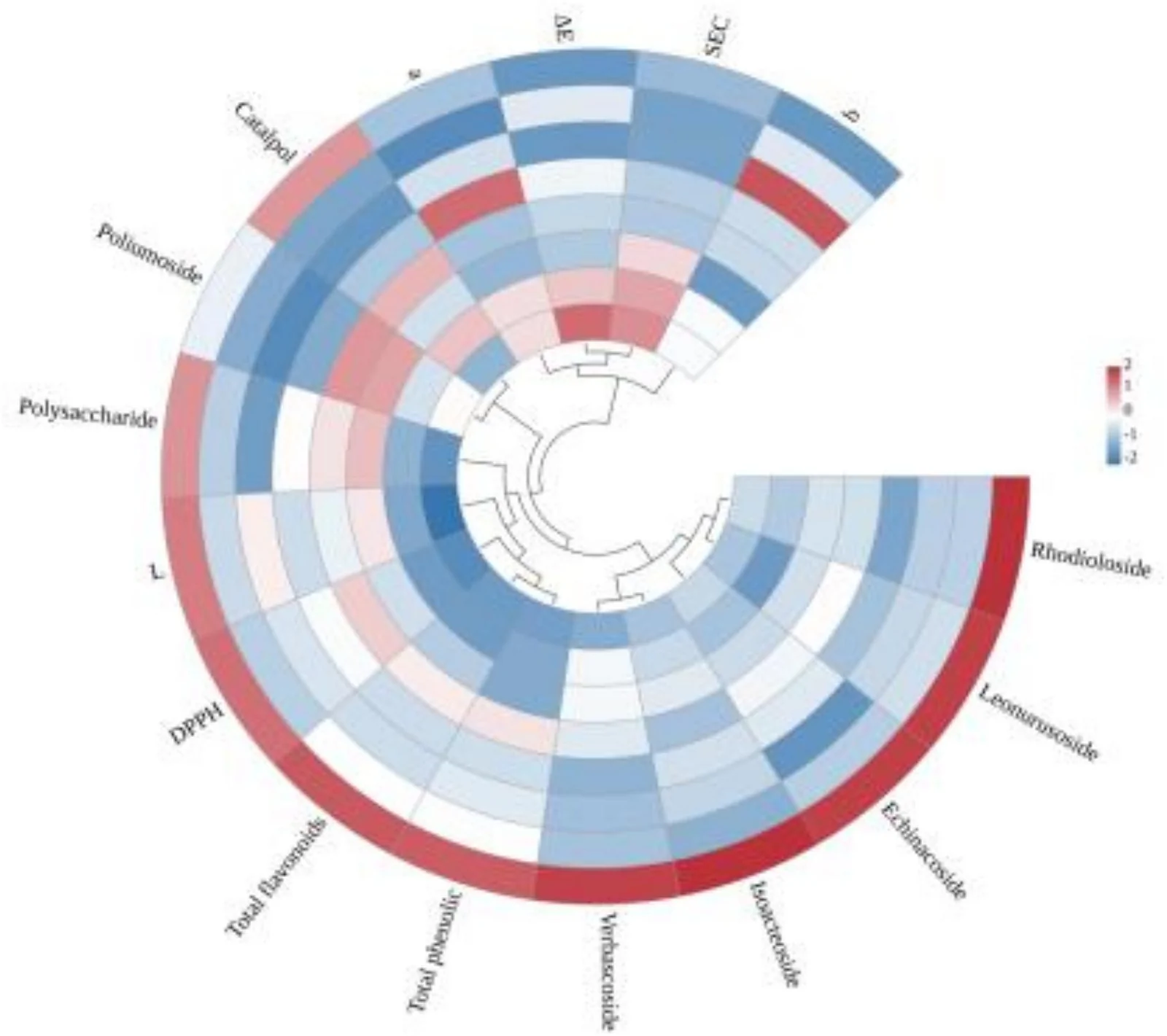
Hierarchical clustering heatmap of Cistanche slices under different drying treatments.

### 3.11 Principal component analysis (PCA)

PCA was performed to further identify the dominant factors responsible for differences among drying treatments (Fig. 11). The first two principal components explained 78.42% of the total variance, indicating that the two-dimensional PCA plot adequately reflected the main differences among treatments in terms of quality, bioactive compounds, color, and energy-related indicators. According to the loading plot, PC1 was mainly positively associated with bioactive compounds and quality indicators. SEC, ΔE, and a* were mainly located in the negative direction of PC1 or away from the cluster of bioactive compounds, indicating that higher energy consumption and greater color difference were opposite to the overall functional-quality characteristics. In the score plot, TFU was located far along the positive direction of PC1 and was clearly separated from the other treatments. This indicates that TFU was most strongly associated with higher measured levels of bioactive compounds, antioxidant capacity, and overall quality improvement. HA and SFU1 were located in the negative direction of PC1 and the positive direction of PC2, indicating weaker association with active-quality indicators and stronger association with unfavorable indicators such as SEC and color deviation. This suggests that hot-air drying alone and 25 kHz single-frequency ultrasound were insufficient to effectively improve the overall quality of dried Cistanche slices. Compared with HA and SFU1, SFU2 and SFU3 were closer to the positive direction of PC1, indicating that sample quality gradually improved with changes in SFU conditions. Although DFU1 and DFU2 had shorter drying times and lower SEC, they did not approach the active-quality loading cluster, indicating that strong drying intensification does not necessarily lead to optimal quality retention. DFU3 was closer to the positive direction of PC1, consistent with its higher antioxidant capacity and higher contents of some active compounds. Therefore, among the dual-frequency treatments, DFU3 was more favorable for quality retention, whereas DFU1 and DFU2 were more closely associated with energy efficiency.

**Fig. 11:**
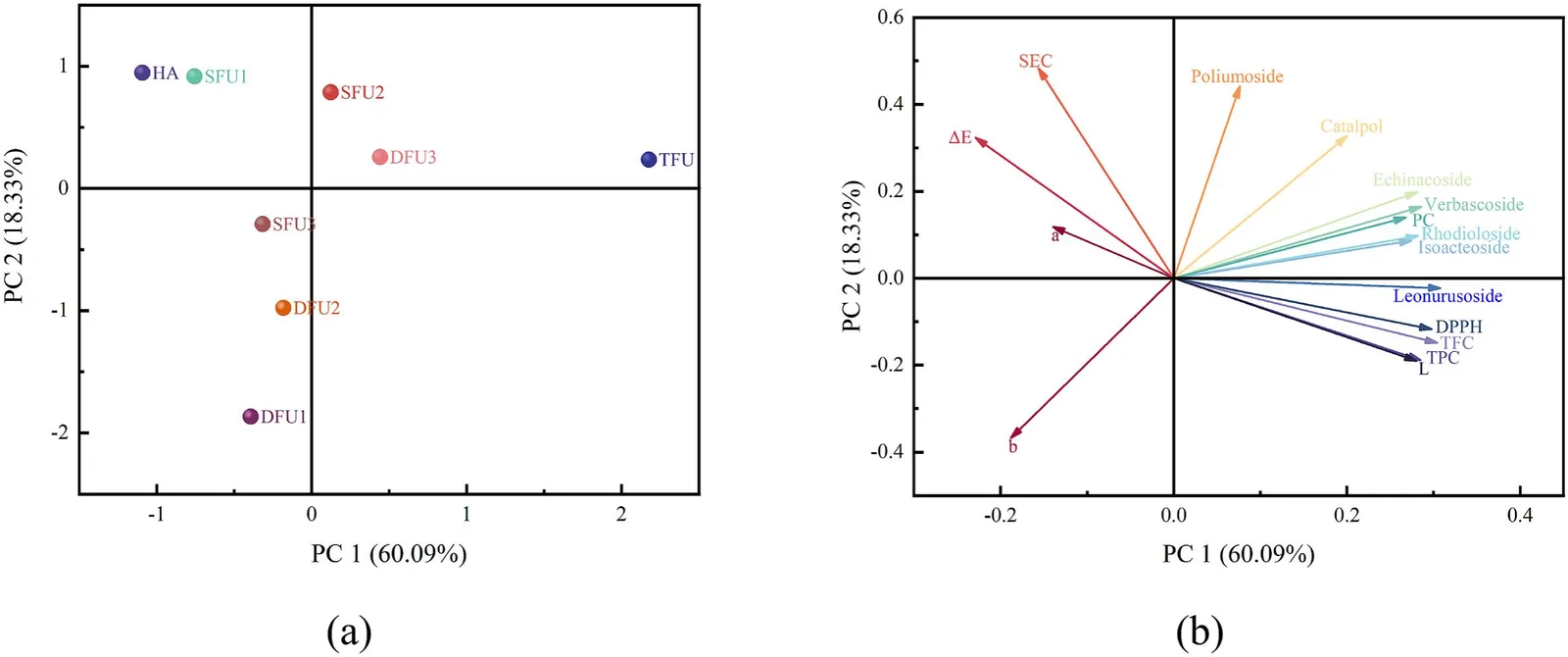
PCA of Cistanche slices subjected to different drying treatments (a) score plot and (b) loading plot.

## 4 Conclusions

This study systematically compared the effects of synchronous SFU, DFU, and TFU-assisted HA on the drying efficiency, energy consumption, quality attributes, characteristic bioactive compounds, and microstructural changes in Cistanche slices. These findings demonstrate that ultrasound-assisted HA facilitated moisture transport within the slices, leading to shorter drying times and lower specific energy consumption. Compared with HA, SFU shortened drying time by 12.50–31.25%, whereas DFU and TFU produced stronger intensification effects. DFU1, DFU2, and TFU all reduced the drying time by 43.75%. Under the present laboratory-scale conditions, DFU2 exhibited the lowest SEC among the tested treatments, although the absolute SEC values obtained at the 120 g loading scale should not be directly extrapolated to industrial operation. Different ultrasound frequency combinations significantly affected quality retention in Cistanche. TFU exhibited higher measured contents in TPC, TFC, PC, and DPPH radical scavenging activity, while also exhibiting the lowest total color difference. This indicates that TFU improved the functional quality and color stability of dried Cistanche. The active-compound results further showed that TFU favored the retention or release of most phenylethanoid glycosides and iridoid glycosides. However, poliumoside was highest in the DFU3 group, indicating that different bioactive compounds responded differently to ultrasound frequency combinations. SEM observations showed that ultrasound produced a more open and porous tissue structure, consistent with the enhanced apparent moisture transport. Multivariate analysis further distinguished the treatment profiles, with TFU more closely associated with most quality-related indicators and DFU2 with energy efficiency. These results indicate that increasing the number of ultrasound frequencies did not produce uniform improvements across all responses; instead, different frequency modes favored different processing objectives. These findings provide practical guidance for applying MFU-assisted HA to the efficient and quality-preserving processing of medicinal and edible plant materials. However, the present study was conducted at laboratory scale under fixed drying conditions, and the spatial acoustic-field distribution and actual acoustic energy delivered to the samples were not directly characterized. In addition, the stability of characteristic phytochemicals should be carefully considered because excessive or spatially non-uniform ultrasonic exposure may adversely affect sensitive bioactive constituents. During scale-up, acoustic attenuation and spatial non-uniformity caused by increased chamber dimensions and material loading may become important constraints. Future studies should therefore integrate acoustic-field characterization with optimization of transducer arrangement, frequency configuration, and power density, followed by pilot-scale validation.

## CRediT authorship contribution statement

**Bowen Wu:** Writing – original draft, Visualization, Methodology, Investigation, Formal analysis, Data curation, Conceptualization. **Xiaopeng Huang:** Writing – review & editing, Supervision, Resources, Methodology, Funding acquisition, Conceptualization. **Guojun Ma:** Supervision, Methodology, Conceptualization. **Fangxin Wan:** Writing – review & editing, Methodology. **Zepeng Zang:** Supervision, Methodology, Conceptualization. **Yanrui Xu:** Supervision, Conceptualization.

## Declaration of competing interest

The authors declare that they have no known competing financial interests or personal relationships that could have appeared to influence the work reported in this paper.
